# Large Depth-of-Field Integral Microscopy by Use of a Liquid Lens

**DOI:** 10.3390/s18103383

**Published:** 2018-10-10

**Authors:** Anabel Llavador, Gabriele Scrofani, Genaro Saavedra, Manuel Martinez-Corral

**Affiliations:** 3D Imaging and Display Laboratory, Department of Optics, University of Valencia, Burjassot, Valencia E-46100, Spain; scrofani@uv.es (G.S.); genaro.saavedra@uv.es (G.S.); manuel.martinez@uv.es (M.M.-C.)

**Keywords:** three-dimensional image acquisition, three-dimensional microscopy, three-dimensional image processing

## Abstract

Integral microscopy is a 3D imaging technique that permits the recording of spatial and angular information of microscopic samples. From this information it is possible to calculate a collection of orthographic views with full parallax and to refocus computationally, at will, through the 3D specimen. An important drawback of integral microscopy, especially when dealing with thick samples, is the limited depth of field (DOF) of the perspective views. This imposes a significant limitation on the depth range of computationally refocused images. To overcome this problem, we propose here a new method that is based on the insertion, at the pupil plane of the microscope objective, of an electrically controlled liquid lens (LL) whose optical power can be changed by simply tuning the voltage. This new apparatus has the advantage of controlling the axial position of the objective focal plane while keeping constant the essential parameters of the integral microscope, that is, the magnification, the numerical aperture and the amount of parallax. Thus, given a 3D sample, the new microscope can provide a stack of integral images with complementary depth ranges. The fusion of the set of refocused images permits to enlarge the reconstruction range, obtaining images in focus over the whole region.

## 1. Introduction

The acquisition of the 3D information of volumetric objects has been extensively developed during the last few decades. Integral imaging (InI), first proposed by Lipmann [1] in 1908, is a technique that permits the capture of spatial and angular information by means of an array of microlenses. Each microlens provides a different perspective of the object, depending on its position on the array. At present, many applications of InI technique have been developed [2,3,4,5]. One of the most interesting applications is the possibility of recovering in-depth information of a 3D scene by applying conventional algorithms of reconstruction [6,7,8].

Recently, the integral imaging technique has been adapted to microscopy [9]. An integral microscope (IMic) was developed to capture both the spatial and the angular information of a 3D microscopic sample from a single shot. After recording, we can process the information to generate a set of perspective views of the sample and reconstruct the 3D scene by depth-reconstruction algorithms. The main drawback of every integral imaging system, including integral microscopes, is the low spatial resolution of the views, compared with the spatial resolution provided by a conventional microscope. In this sense, many different solutions have been proposed [10,11].

Apart from the loss of resolution, we cannot ignore the restriction imposed by the depth of field (DOF) of the refocusing process. Take into account that the refocusing algorithms can only provide sharp refocused images in planes within the DOF of the perspective views. The DOF limitations are determined by the parameters of the imaging system and cannot be overcome by either optical or computational methods. Therefore, new kinds of solutions are necessary to overcome this problem.

In this sense, in this paper we propose a new technique based on the insertion of a single electrically tunable liquid lens (LL) at the aperture stop (AS) of the integral microscope. An electrically tunable liquid lens is a focusing element composed, essentially, by a plane and a spherical diopter. Based on the electro-wetting technology, the optical power of the spherical diopter can be finely controlled by tuning the voltage applied to the LL [12]. In the last years, LL have been used for many purposes, being one of the most interesting micro-imaging systems due to the possibility of performing fast and non-mechanical refocusing [13,14,15]. A slightly different approach can be found in [16], where the authors use a liquid lens as a vary-focal tube lens (TL). On the other hand, the use of different kinds of dynamic elements has been proposed recently in integral imaging microscopy. For instance, in Ref. [17] the authors propose the replacement of the microlens array (MLA) by a multifocal high-resistance liquid crystal microlens array. On the contrary, in References [18,19], the use of a bifocal polarization-dependent liquid-crystalline polymer microlens array (LCP-MLA) and a bifocal holographic micro lens array, respectively, are proposed. In all cases, the authors achieved an extended working range of the integral imaging microscope. However, there are important differences between such proposals and the one submitted here. The first difference is that integral imaging microscopes are based on the so-called plenoptic 2.0 concept [20] while the IMic is based on the plenoptic 1.0 concept [21]. The second difference is that those proposals are based on the use and control of non-commercially available arrays composed by hundreds of microlenses. However, our system is based on the use of a single, and commercially available, liquid lens. The third advantage is that our proposal preserves the telecentricity of the system, meaning that the magnification of the system does not change along the extended working range. Thus, no additional computational correction of the scale is necessary. An additional and non-negligible advantage is that the 1.0 configuration obtains maximum results from the CCD pixels due to the minimization of the vignetting effect at the sensor plane.

The paper is organized as follows: Section 2 establishes the basic theory of an integral microscope and explains the main drawback in terms of depth of field; Section 3 presents the proposed method to enlarge the reconstruction range; Section 4 shows experimental data to validate the method; and Section 5 presents the main conclusions of our work.

## 2. The Integral Microscope: Configuration and Depth of Field

**Figure 1:**
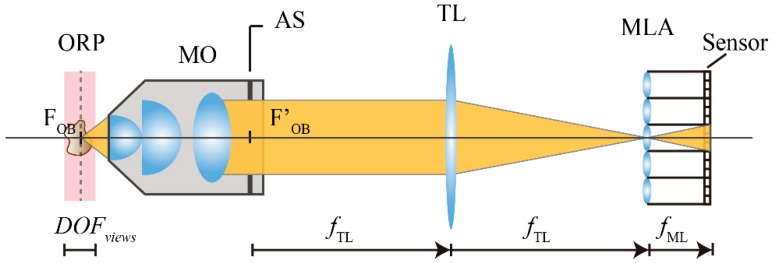
Scheme of an IMic. The MO and the TL are placed in afocal configuration. The MLA is located at the intermediate image plane and the sensor is shifted by a distance of $f_{\mathrm{ML}}$.

Let us start by considering the basic structure of an integral microscope. As it can be seen in Figure 1, the microscope objective (MO) and the tube lens of the host microscope are placed in afocal configuration. An IMic is built by inserting a microlens array at the image plane of the host microscope. The sensor is then shifted by a distance that is equal to the microlenses focal length, $f_{\mathrm{ML}}$. With this configuration, the camera sensor records a set of micro images, as many as the number of microlenses in the array, which contains the spatial and angular information of a 3D microscopic sample. To make proper use of the pixels of the camera and thus to avoid any overlapping between the microimages, it is important to match the numerical aperture of the MO, expressed at the image side ${({NA}^{\prime }}_{\mathrm{MO}}$), and the numerical aperture of the microlenses (${NA}_{\mathrm{ML}})$. This condition can be written as

(1) $${{NA}^{\prime }}_{\mathrm{MO}}=\frac{NA_{\mathrm{MO}}}{M}=NA_{\mathrm{ML}}.$$

being ${M=f}_{\mathrm{TL}}{/f}_{\mathrm{OB}}$ the lateral magnification of the microscope.

One of the most important applications of the integral imaging technology is the possibility of computing a set of perspective views of the object from the information provided by the integral image. These perspective views, called sub-images, allow us to perform a depth reconstruction of the 3D scene by applying conventional algorithms. From a computational point of view, the number of reconstruction planes is only limited by the number of pixels within each sub-image. However, the refocusing capability of typical algorithms is restricted to those regions of the scene that appear sharp in the perspective views. In other words, the depth of field of the sub-images imposes a strong limitation on the effective number of planes that we can generate. This is especially important when working in integral microscopy due to the much reduced DOF of the perspective views. Taking into account the conventional procedure to extract the different views from the integral image [22], the DOF of each of these sub-images can be expressed as [23]:

(2) $$DOF_{views}=\frac{\lambda }{2NA_{\mathrm{MO}}^{2}}\left(2+\mu^{2}\right).$$

In the last equation, λ is the wavelength of the illumination and μ is a dimensionless parameter that relates the size of the microlenses with the host point spread fuction (PSF), $\rho_{\mathrm{host}}$, i.e.,:

(3) $$\mu =\frac{p}{M\rho_{host}},$$

with

(4) $$\rho_{host}=\frac{\lambda }{2NA_{\mathrm{MO}}}.$$

It is important to note that all the parts of the 3D sample that fall outside the region defined by Equation (2), will appear blurred in the reconstruction. As mentioned above, this means that although computationally there is a relatively high number of reconstruction planes, the number of planes wherein the information is in focus is limited by Equation (2).

## 3. Enlarged the Reconstruction Range by Using a Liquid Lens: Proposed Method

**Figure 2:**
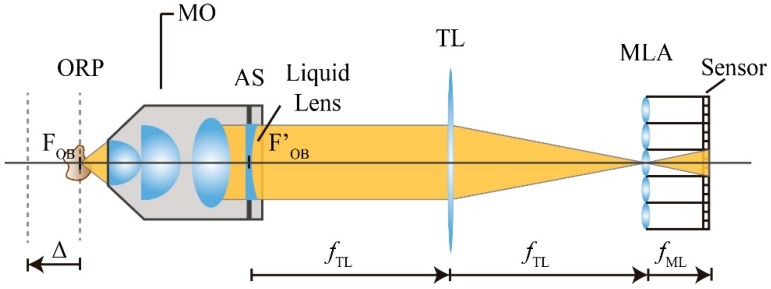
Insertion of a liquid lens at the AS of the MO. With this configuration, a displacement of the object reference plan (ORP) is achieved.

To increase the number of effective planes of reconstruction, we propose a method based on the insertion of a liquid lens (LL) with tunable focus at the aperture stop of the MO (see Figure 2).

To understand how this new setup works, we can consider the MO and the LL as a two-lens coupled-system. From Geometrical Optics, it is known that every optical system, no matter how complex, can be expressed by a pair of principal and focal planes, calculated from the parameters that compose each individual system. In our case, the LL is placed just at the back focal plane (BFP) of the MO; thus, it is easy to demonstrate that the focal length of the equivalent system, $f_{T}$, is

(5) $$f_{T}=f_{\mathrm{OB}}.$$

From Equation (5) we can see that the focal length of the equivalent system equals the focal length of the MO. Moreover, the insertion of a second lens does not modify the position of the BFP of the global system, ${F^{\prime }}_{T}$. On the other hand, the coupling of the two lenses produces a shifting on the position of the front focal plane (FFP) of the equivalent system. This displacement Δ can be expressed as

(6) $$\Delta =\frac{f_{\mathrm{OB}}{}^{2}}{f_{\mathrm{LL}}}=P_{\mathrm{LL}}f_{\mathrm{OB}}{}^{2},$$

being *P*_LL_ the optical power of the liquid lens, $P_{\mathrm{LL}}=1/f_{\mathrm{LL}}$. Note from Equation (6) that we can obtain positive and negative displacements of the FFP, by considering different values of *P*_LL_.

Thus, we can affirm that by inserting a LL at the BFP of a microscope objective (i.e., its pupil plane), it is possible to change the position of the object reference plane of the microscope, while the other parameters of the system, like magnification and resolution power, remain invariant. Note also that the insertion of the liquid lens at the proper plane does not change the telecentricity of the original system which is important in microscopy. Moreover, we can affirm that the insertion of a LL at the correct plane does not change the PSF of the system, as demonstrated in Ref. [15].

By taking advantage of the characteristics of this new setup, we propose to capture a collection of integral images, each one corresponding to a different axial position of the ORP of the microscope. From the combination of the reconstructions obtained from this set of integral images, we are able to extend the total range of reconstruction.

## 4. Experimental Results

To validate our method, we performed the following experiment in our laboratory. We implemented an IMic like the one shown in Figure 2, with a MO magnification of 50× and numerical aperture of *NA* = 0.55, and a TL with focal length of $f_{\mathrm{TL}}=100$ mm. The MLA had a pitch of p = 110 μm and a numerical aperture ${\mathrm{NA}}_{\mathrm{ML}}$ = 0.01 (MLA #12-1192-106-000, from SUSS MicroOptics, Hauterive, Switzerland). To capture the images, we used a digital camera (Canon EOS 450D) assembled with a macro objective 1:1. To perform the displacement of the ORP we used a liquid lens with a diameter of $\phi_{\mathrm{LL}}=3.5$ mm. The LL was a commercial one manufactured by Varioptic, Lion, France (ARCTIC 39N0); it is based on the electrowetting principle and it is composed by two liquids with the same density but different refractive index. When a voltage is applied, the curvature of the interface changes. In the linear range, the relation between the optical power and the applied voltage is given by:

(7) $$P_{LL}(T)=S(T)[V-V_{0}(T)],$$

where T is the environmental temperature measured in Celsius (°C). Under our experimental conditions (T=20 °C), S(25)=1.068 (m·V)^−1^ and $V_{0}(25)=41.1$ V. 

**Figure 3:**
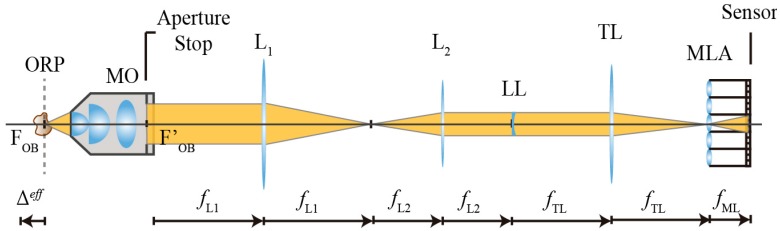
Scheme of an IMic as implemented in the laboratory. We used a relay system composed by two lenses of focal lengths *f*_L1_ = 150 mm and *f*_L2_ = 75 mm, respectively, and disposed of in afocal configuration. The LL is then placed at the back focal plane of L_2_.

As it was not possible to physically access the pupil plane of the MO, we needed to introduce a relay system in order to place the LL at the correct plane. The relay was composed by two lenses: L_1_ and L_2_ placed in afocal configuration as we can see in the scheme shown in Figure 3. The exit pupil of the MO had a diameter of $\phi_{P}=7.2$ mm, whilst the diameter of the liquid lens was $\phi_{\mathrm{LL}}=3.5$ mm. In order to match both diameters as much as possible we chose a combination of lenses so that the lateral magnification of the relay was $M_{R}=0.5$. In particular, we used two lenses with $f_{L1}=150$ mm and $f_{L2}=75$ mm, respectively.

It is important to note that the LL is not inserted directly at the aperture stop of the MO but in a conjugated plane, so Equation (6) has to be modified to take into account the effective optical power induced at the liquid lens ($P_{\mathrm{LL}}^{eff}$). In this way, the displacement of the ORP is now expressed by:

(8) $$\Delta^{eff}=P_{\mathrm{LL}}^{eff}f_{\mathrm{OB}}^{2},$$

with

(9) $$P_{\mathrm{LL}}^{eff}=P_{\mathrm{LL}}M_{R}^{2}.$$

**Figure 4:**
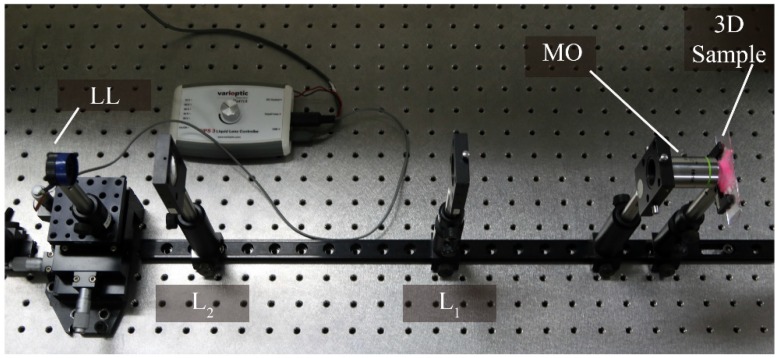
Details of the experimental setup.

The 3D object used in the experiment was composed by fluorescent cotton fibers. The sample was illuminated with a laser of wavelength λ = 532 nm. In Figure 4 we show a picture of the details of the experimental setup, from the 3D sample to the LL.

With our integral microscope we captured a total of 10 integral images each of which corresponded to a different position of the ORP. To change the axial position of the ORP, we applied different voltages to the LL, from V = 38.0 V to V = 56.9 V, with increments of 2.1 V. In Table 1 we show, for each value of the applied voltage (V), the optical power (*P*_LL_) induced in the LL and the corresponding displacement of the ORP, Δ_eff_, calculated with Equations (7)–(9). From these values, it can be seen that the displacement between two consecutive captures is 9 µm, which corresponds to approximately DOF/2, as we will see later.

**Figure 5:**
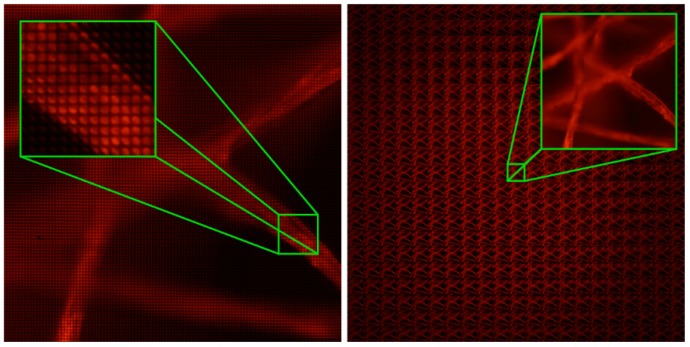
(**a**) Integral image registered with the IMic. The image is composed by 111 × 111 micro-images. (**b**) Set of 21 × 21 sub-images computed from (**a**).

To illustrate the main drawback of integral microscopy in terms of the reconstruction range let us consider first the integral image captured with our IMic when a voltage of V = 48.5 V is applied (see Figure 5a). The integral image is composed of 111 × 111 micro-images, with 21 × 21 pixels each. From this integral image we can compute a collection of perspective views by applying the conventional algorithms. In Video S1 we show a video with many of these views. The spatial resolution of the perspective views is 4.4 µm [11]. From the set of sub-images shown in Figure 5b it is easy to note that many regions of the 3D sample are out of focus. As a matter of fact, the DOF is not very extended. We can calculate it using Equation (2) whilst taking into account the parameters of the system, which we can see is around 17 μm (for an emission wavelength of 630 nm).

**Figure 6:**
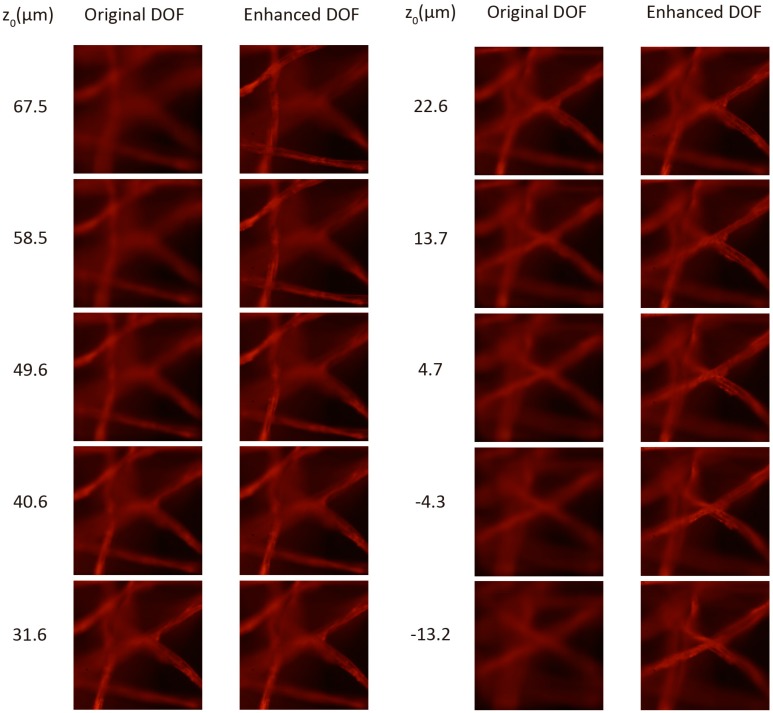
Comparison of the depth reconstruction by the conventional method (**left**) and with the proposed method (**right**).

The depth reconstruction obtained from the single capture shown in Figure 5a can be seen in the left column of Figure 6 together with the corresponding axial distance $z_{0}$. As expected, we can check that the DOF is limited and consequently, the small region of sharp reconstruction. In the right column of Figure 6 we show the reconstruction over the same range that is in the left column, after combining the individual planes obtained from the stack of integral images captured as we explained before. Note that, while in the left column only the plane corresponding to $z_{0}=31.6$ μm is in focus, the proposed method allows us to enlarge the reconstruction range, thereby obtaining images with high sharpness over the whole range (see Video S2).

**Figure 7:**
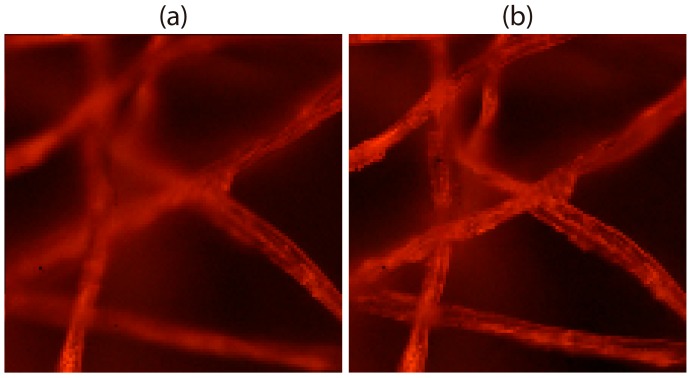
Comparison of the depth of field in perspective views with (**a**) conventional integral microscopy, and (**b**) the proposed method. Note in the last case how the range in which the sample appears sharp is increased.

Finally, we can compare this improvement in the DOF in terms of the perspective views. In Figure 7a, we show the central view of the integral microscope extracted from the sub-images shown in Figure 5b. On the other hand, Figure 7b shows the same central view of the 3D sample, but only after combining the multiple integral images registered. Note that, in this case, the DOF of the perspective views is also enlarged.

## 5. Conclusions

In this paper we have proposed a new integral microscope with strongly enlarged depth refocusing range. The insertion of a LL at the pupil plane of the microscope objective, permits us to change the axial position of the object plane and therefore register a stack of integral images. The combination of the individual reconstructions provides an extended reconstruction range with sharp images over the whole range, in comparison to the conventional realization of integral microscopy where the information is blurred outside the region defined by the depth of field of the perspective views. Experimental data is shown to validate our method, which has many advantages with respect other works, as previously reported.

## Figures and Tables

**Table 1:** Optical power in the LL and effective displacement of the ORP induced while changing the voltage.

V (V)	*P*_LL_ (m^−1^)	Δ_eff_ (μm)
38.0	–3.3	–13.2
40.1	–1.1	–4.3
42.2	1.2	4.7
44.3	3.4	13.7
46.4	5.7	22.6
48.5	7.9	31.6
50.6	10.1	40.6
52.7	12.4	49.6
54.8	14.6	58.5
56.9	16.9	67.5

## Supplementary Materials

The following are available online at http://www.mdpi.com/1424-8220/18/10/3383/s1.
